# Ontogeny and colonization of embryonic border-associated macrophages and their role in neurodevelopment

**DOI:** 10.3389/fncel.2025.1677414

**Published:** 2025-10-30

**Authors:** Ashley M. Otero, Adrienne M. Antonson

**Affiliations:** 1 Neuroscience Program, University of Illinois Urbana-Champaign, Urbana, IL, United States; 2 Department of Animal Sciences, University of Illinois Urbana-Champaign, Urbana, IL, United States

**Keywords:** border-associated macrophages, microglia, embryonic brain development, neuroimmune cells, maternal immune activation, neurodevelopmental disorders

## Abstract

Border-associated macrophages (BAMs) are tissue-resident macrophages in the central nervous system (CNS) that originate from yolk sac progenitors during primitive hematopoiesis. While much is known about their parenchymal counterparts, microglia, recent evidence indicates that BAMs also play roles in neurodevelopment. Located at CNS interfaces such as the meninges, choroid plexus, and perivascular space, BAMs facilitate immune surveillance, vascular modeling, debris clearance, and cerebrospinal fluid dynamics. Despite their strategic location, BAMs have historically been understudied in developmental contexts. This mini review covers their embryonic origins, regional diversification, and functional roles as development progresses. Offering new insights, we consider BAMs in the context of neurodevelopmental disorders (NDDs). Recent findings from maternal immune activation (MIA) studies suggest that fetal BAMs may contribute to aberrant cortical development through altered inflammatory signaling. We propose that, like microglia, BAMs may play previously unappreciated roles in shaping the developmental trajectory of the brain. To aid future research, we also review current tools for studying BAMs *in vivo* and *in vitro*, including new transgenic lines and organoid-based approaches. These tools will be critical for dissecting the molecular functions of BAMs during healthy and disordered development. Understanding BAM biology in early life may reveal novel mechanisms underlying NDDs and inform therapeutic strategies targeting brain–immune interfaces.

## 1 Introduction

Macrophages are innate immune cells that specialize in the detection and phagocytosis of pathogens. They can be classified into tissue-resident macrophages, which originate during embryogenesis, and circulating monocyte-derived macrophages, which develop postnatally. Parenchymal microglia and non-parenchymal border-associated macrophages (BAMs) are the tissue-resident macrophages of the central nervous system (CNS). Yolk-sac-derived myeloid progenitors migrate into the brain during embryogenesis and become brain-resident macrophages (Prinz and Priller, 2014; Ginhoux et al., 2010). Microglia infiltrate the brain parenchyma radially from the meninges and ventricles (Swinnen et al., 2013) and are the only glial cells present at the onset of neurogenesis, allowing them to support early neuronal development (Reemst et al., 2016). During fetal brain development, microglia promote cortical neural precursor cell (NPC) proliferation and migration (Antony et al., 2011; Shigemoto-Mogami et al., 2014), phagocytose excess NPCs in the cortex (Cunningham et al., 2013), influence interneuron wiring and positioning (Squarzoni et al., 2014; Thion et al., 2019), and assist in axonal fasciculation (Pont-Lezica et al., 2014). Microglia continue to shape the brain postnatally by pruning synapses (Paolicelli et al., 2011; Zhan et al., 2014) and inducing dendritic spine formation (Miyamoto et al., 2016). Disruptions in any of these processes can be detrimental, potentially leading to neurodevelopmental disorder (NDD) phenotypes in offspring. Animal models of maternal immune activation (MIA), induced by viral infections and immunostimulants, have shown core NDD-related pathologies in offspring such as abnormal cortical development (Choi et al., 2016; Otero et al., 2025; Carpentier et al., 2013; Ben-Reuven and Reiner, 2021; Canales et al., 2021), dysregulated interneuron wiring (Meyer et al., 2008; Canetta et al., 2016; Shin Yim et al., 2017; Vasistha et al., 2020), and altered synaptic pruning (Fernández de Cossío et al., 2017). Notably, these neuropathologies all rely on microglia-mediated activities. Indeed, several of these studies directly implicate microglia in inflammation-driven brain abnormalities (Pont-Lezica et al., 2014; Loayza et al., 2023; Yu D. et al., 2022; Rosin et al., 2021). Less is known about the role of BAMs during embryonic development and how these cells contribute to homeostatic and diseased conditions.

BAMs are ontogenically unique and do not infiltrate the brain like microglia; instead, they reside in peripheral regions such as the choroid plexus, meninges, and perivascular spaces. Their location near the periphery allows for constant immune surveillance and phagocytosis of debris (Dermitzakis et al., 2023; Brioschi et al., 2023; Van Hove et al., 2019). One study demonstrates that embryonic choroid plexus BAMs are impacted by prenatal inflammation, and it is hypothesized that these cells could indirectly contribute to aberrant cortical development, although the exact mechanisms are unknown (Cui et al., 2020). This mini review aims to summarize our current knowledge on BAMs, especially during embryonic development, while also addressing the limited understanding of their roles during early immune challenges.

## 2 Developmental origins of brain-resident macrophages

**FIGURE 1:**
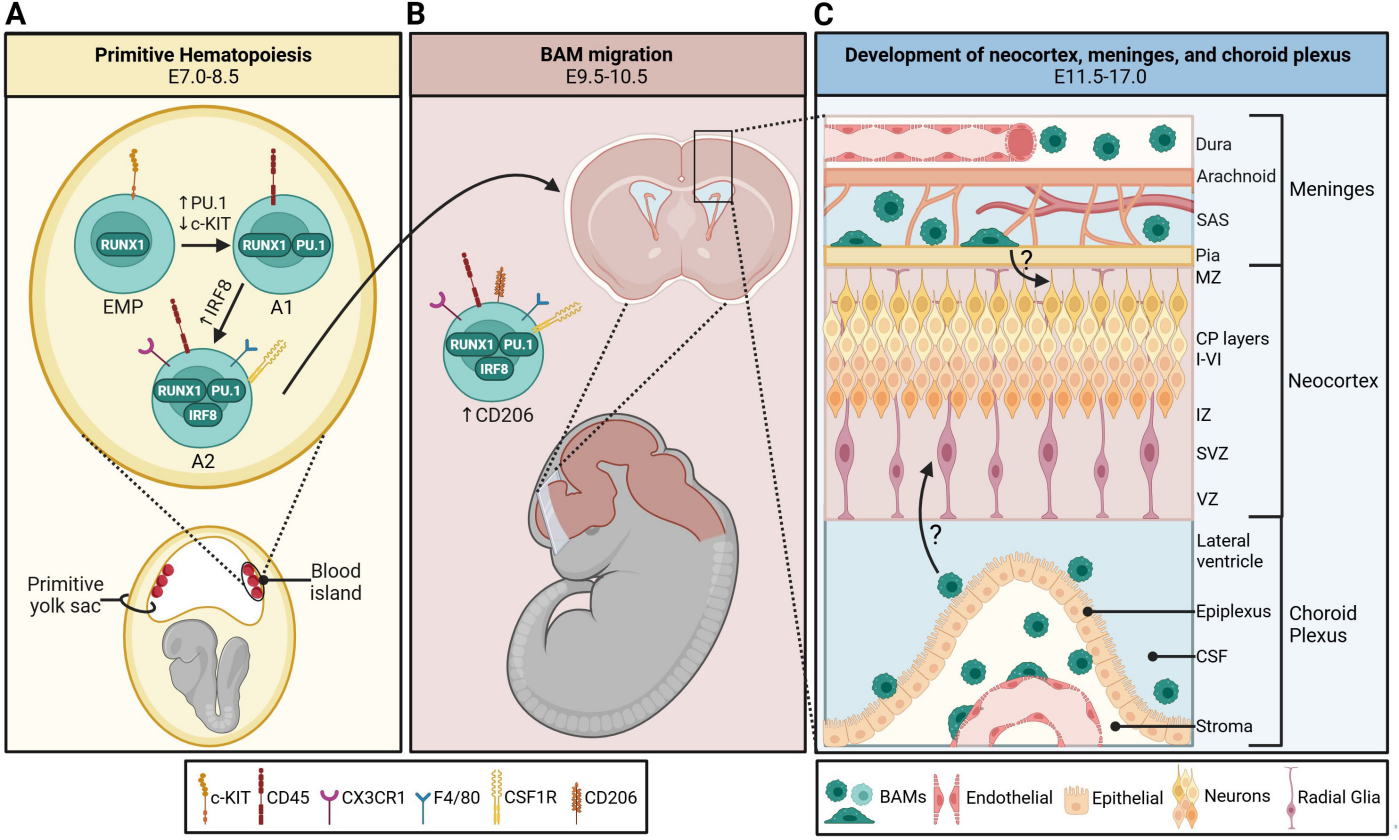
Development and colonization of border-associated macrophages (BAMs) in the embryonic mouse brain. **(A)** BAMs originate from c-KIT^+^ RUNX1^+^ erythromyeloid progenitors (EMPs) in the primitive yolk sac blood island starting on embryonic day (E)7.0. They transition into A1 progenitors with the acquisition of macrophage-specific transcription factor PU.1 and surface marker CSF1R. These cells become A2 progenitors once they upregulate IRF8, CD45, and CX3CR1. **(B)** BAM A2 progenitors upregulate CD206 and migrate to the developing fetal brain starting on E9.5. **(C)** CD206^+^ BAMs take residence in the developing meninges and choroid plexus between E11.5-17.0, and the six cortical layers develop concurrently. It is currently unknown how extra-parenchymal embryonic BAMs may contribute to neocortical development. CP, cortical plate; CSF, cerebrospinal fluid; IZ, intermediate zone; MZ, marginal zone; SAS, subarachnoid space; SVZ, subventricular zone; VZ, ventricular zone. Schematic was generated with BioRender.com.

CNS myeloid cell development is hypothesized to be conserved across species (Penati et al., 2025). However, evolutionary differences between humans and mice require different approaches for studying human macrophages (Bao et al., 2024). Based on the research available, we focus on the mouse developmental timeline in this review. Two waves of hematopoiesis constitute embryonic hematopoietic development. Definitive hematopoiesis in mice occurs in the aorta–gonad–mesonephros region and fetal liver around embryonic day (E)10.5 and E12.5, respectively (Prinz and Priller, 2014). These hematopoietic stem cells differentiate into myeloid progenitors, which further differentiate into self-renewing tissue-resident macrophages, such as Kupffer cells of the liver (Epelman et al., 2014). At the turn of the 21st century, a transient period of embryonic hematopoiesis was discovered (Palis and Yoder, 2001). This wave, termed primitive hematopoiesis, occurs around E7.5 to E8 and gives rise to erythromyeloid progenitor (EMP) cells in the yolk sac blood island. Some epidermal Langerhans cells and all brain macrophage cells are yolk-sac-derived (Sheng et al., 2015). Uncommitted EMPs are identified by the expression of surface marker tyrosine-kinase protein (c-KIT) and runt-related transcription factor 1 (RUNX1) (Ginhoux et al., 2010; Zusso et al., 2012; Kierdorf et al., 2013; Utz et al., 2020). RUNX1^+^c-KIT^+^ progenitors downregulate c-KIT and acquire transcription factor PU.1, which is required for commitment to the CNS macrophage lineage (Kierdorf et al., 2013; Van Hove et al., 2019). Immature macrophage A1 progenitors express surface marker CD45^+^ and upregulate transcription factor IRF8 along with surface markers F4/80, fractalkine receptor (CX3CR1), and colony-stimulating factor 1 receptor (CSF1R), allowing them to develop into A2 macrophage progenitors that can migrate to the developing brain starting at E9.5 (Ginhoux et al., 2010; Kierdorf et al., 2013; Stremmel et al., 2018; Gomez Perdiguero et al., 2015; Utz et al., 2020; Figure 1A). A fate-mapping study by Utz et al. (2020) demonstrated that brain macrophage populations begin to separate as early as E10.5 in the yolk sac and brain with the expression of BAM-specific surface marker, CD206 (Utz et al., 2020). Their paper postulates that early differentiation of CD206^–^ macrophages into CD206^+^ macrophages indicates that BAMs and microglia are separate populations in the yolk sac (Utz et al., 2020). However, other fate-mapping studies contradict this, stating that CD206^+^ cells can infiltrate the neocortex from the meninges/ventricles and become CD206^–^ microglia (Masuda et al., 2022; Hattori et al., 2023). It is possible that both postulates are true depending on the context and stage of development. Interestingly, cells double-positive for CD206 and P2RY12 (a microglia-specific marker) were transiently observed in both human and rodent CNS tissue at Carnegie stage (CS)12 and E10.5, respectively, indicating a less clear lineage separation (Wu et al., 2025). Once in the brain, microglia migrate from the ventricles and meninges into the parenchyma (Mosser et al., 2017). BAMs, however, do not infiltrate the parenchyma and rather take residence in the brain’s peripheral regions (Figure 1B). The microenvironment of these regions produces ontogenetically unique BAM subtypes (Van Hove et al., 2019; Masuda et al., 2022). In the following sections, we outline regional specificity of these cells and provide an overview of what is (and is not yet) known about their functional role in early development.

## 3 Regional specificity determines BAM ontogeny and function in early life

Fate-mapping studies indicate that perinatal BAMs are initially homogenous populations that develop regional specificity after birth (Utz et al., 2020; Van Hove et al., 2019; Masuda et al., 2022). Conserved genes include Mrc1 (the gene encoding CD206), Stab1, Pf4, Ms4a7, Lyve1, Siglec1, and Cd38 (Brioschi et al., 2023; Utz et al., 2020; Mrdjen et al., 2018; Jordão et al., 2019). After birth, BAM subtypes emerge, and transcriptional divergence correlates with functional differences. These subtypes can be identified by major histocompatibility complex class II (MHCII), lymphatic vessel endothelial hyaluronan receptor 1 (LYVE1), and CD38 expression levels, as well as possible replenishment from CCR2^+^ blood monocytes (Van Hove et al., 2019; Brioschi et al., 2023; Mrdjen et al., 2018). For a thorough review on BAM regional specificity, see Zhan et al. (2024) (Zhan et al., 2024).

### 3.1 Meningeal BAMs

The meninges form a protective barrier around the brain and consist of the dura mater, arachnoid mater, and pia mater. The primary meninx develops from the mesenchymal sheath around E9.5 in mice after the closure of the neural tube (Dasgupta and Jeong, 2019). About a day later, meningeal vasculature begins to develop (McLone and Bondareff, 1975). By E13, the primary meninx differentiates into the dermal layer, calvarial layer, and primordial meninges, which eventually develop into the dermis, skull, and meninges, respectively (Dasgupta and Jeong, 2019). Between E14-16, cavitation in the leptomeninges (arachnoid and pia mater) generates the cerebrospinal fluid (CSF)-filled subarachnoid space (SAS) (McLone and Bondareff, 1975). By E17, the meningeal blood-CSF barrier is functional (McLone and Bondareff, 1975; Derk et al., 2023). Embryonic meningeal BAMs, which start populating these spaces as early as E11.5, split into two ontogenically unique populations by postnatal day (P)21 based on their location in the dura or leptomeninges (Van Hove et al., 2019).

The dura mater, the outermost layer closest to the skull, contains fenestrated capillaries and lymphatic vessels that drain into the cervical lymph nodes (Louveau et al., 2015). The dural venous sinus within the dura mater collects CSF waste from arachnoid projections (Louveau et al., 2015). BAMs residing near the dural sinuses are exposed to metabolic waste and potential pathogens; therefore, they require the ability to present antigens to trafficked or patrolling T cells (Min et al., 2024; Rustenhoven et al., 2021). While some dural BAMs retain their embryonic origin, many are replaced postnatally by circulating MHCII^+^CCR2^+^ monocytes (Van Hove et al., 2019; Mrdjen et al., 2018; Rebejac et al., 2022), with potential replenishment from skull bone marrow monocytes (Cugurra et al., 2021). The importance of MHCII^+^ dural BAMs is demonstrated by Rebejac et al. (2022), who found these macrophages essential for clearing lymphocytic choriomeningitis virus (LCMV) (Rebejac et al., 2022). However, the antigen presentation capabilities of these cells remain poorly understood (Zhan et al., 2024). We do know that their anti- and pro-inflammatory signaling aids in pathogen clearance. For instance, dural BAMs’ inflammatory responses, such as interferon-gamma receptor (IFNAR)-signaling, allow them to clear peripheral infections (Rebejac et al., 2022). Resident dural BAMs were also necessary for clearing *Trypanosoma brucei* infection via pro-inflammatory cytokine signaling and recruitment of peripheral immune cells (De Vlaminck et al., 2022).

Leptomeningeal BAMs reside in the CSF-filled SAS, which is vascularized with non-fenestrated blood vessels containing tight junction proteins (Zhan et al., 2024). Due to this selectively semi-permeable barrier, BAMs here mostly retain their embryonic origins and can self-renew (Goldmann et al., 2016; Van Hove et al., 2019). A recent study suggests that certain disease states could contribute to partial replacement of these cells by CCR2^+^ monocytes (Wang et al., 2024). This idea is supported by upregulated MHCII-related gene expression within these cells during experimental autoimmune encephalomyelitis (EAE) (Jordão et al., 2019). Leptomeningeal BAMs homeostatically express hemoglobin-haptoglobin scavenger receptor CD163 (Van Hove et al., 2019; Kearns et al., 2023) and LYVE1 (Van Hove et al., 2019; Masuda et al., 2022; Mrdjen et al., 2018), indicating their roles in scavenging/phagocytosis of debris and in arterial motion/CSF flow dynamics, respectively (Drieu et al., 2022; Van Hove et al., 2019).

The role of meningeal BAM subsets in health and disease is an active area of research. Da Mesquita and Rua’s (2024) review summarizes the current knowledge of these cells during adulthood, aging, and disease, and also outlines potential directions for future studies (Da Mesquita and Rua, 2024).

### 3.2 Perivascular BAMs

SAS and subpial blood vessels penetrate into the brain parenchyma (Yu L. et al., 2022). The perivascular space (PVS), also known as the Virchow-Robin space, is the fluid-filled space between these blood vessels and the parenchymal basement membrane, which is overlayed by glia limitans (Ineichen et al., 2022). This structure facilitates CSF flow through the parenchyma and clearance of interstitial fluid. Because of their constant exposure to metabolic waste, cells like perivascular macrophages (PVMs) are necessary for immune surveillance and phagocytosis of debris in the PVS (Zhan et al., 2025).

The PVS forms during the first three postnatal weeks in rodents, around the time of cerebrovascular development (Karam et al., 2022; Coelho-Santos and Shih, 2020). Therefore, PVM populations are postulated to be seeded by perinatal leptomeningeal BAMs (Masuda et al., 2022). Like leptomeningeal BAMs, PVMs self-renew (Jordão et al., 2019; Siret et al., 2022) and upregulate LYVE1 (Masuda et al., 2022; Drieu et al., 2022; Siret et al., 2022; Mrdjen et al., 2018) and CD163 (Van Hove et al., 2019; Siret et al., 2022) expression. Unlike meningeal BAMs, PVMs depend on integrin and vascular smooth muscle cell signaling for proper establishment in the PVS (Masuda et al., 2022). Additionally, a recent study using parabiosis and skull transplant demonstrated that skull bone marrow-derived monocytes can replenish not only dural BAMs, but also leptomeningeal and perivascular BAMs (Du et al., 2024). This newly identified route of BAM replenishment highlights the role of local bone marrow niches in CNS border immunity.

PVMs are easily identified by their elongated morphology surrounding blood vessels (Zhan et al., 2024). This increased surface area allows PVMs to maintain the blood-brain barrier (BBB) through immune surveillance, clearance of blood-borne pathogens/debris, and potential antigen presentation (Zhan et al., 2025). PVMs also exert proangiogenic effects, as shown in a time-resolved scRNA-seq developmental atlas study (Wang et al., 2023). Postmortem studies in humans suggest that PVMs in healthy brains constitutively express MHCII, much like any other antigen-presenting cells (APCs), to present antigens to CD4^+^ helper T cells (Fabriek et al., 2005). In rodents, however, PVMs only express MHCII during pathological inflammatory states like EAE (Jordão et al., 2019) and 5xFAD (an Alzheimer’s Disease mouse model) (Drieu et al., 2022), with very few PVMs expressing MHCII^+^ under homeostatic conditions (Mrdjen et al., 2018; Siret et al., 2022). A recent review by Zhan et al. (2025) highlights our current understanding of the complex role PVMs play in health and disease.

### 3.3 Choroid plexus BAMs

The choroid plexus (ChP), characterized by an outer epithelial layer surrounding a vascularized stromal core, resides in the ventricles of the brain and produces CSF (Ghersi-Egea et al., 2018). ChP development begins as early as E8.5, coinciding with neural tube closure and dorsal midline formation (Currle et al., 2005). Invagination of the dorsal midline structures (roof plate and neurepithelium), along with organizational cues via Wnt and bone morphogenetic protein (BMP) signaling at E10-10.5 in the telencephalon, diencephalon, and hindbrain, leads to the formation of the choroid plexus epithelium in the lateral, third, and fourth ventricles, respectively (Broom et al., 2012; Kompaníková and Bryja, 2022; Lun et al., 2015). Dorsal midline folding also results in the development of progenitor domains: the cortical hem in the telencephalon and the rhombic lip in the hindbrain (Kompaníková and Bryja, 2022). Signals from these progenitor regions provide cues to developing ChP epithelial cells, triggering morphological changes from E11.5-14.5 (Currle et al., 2005; Broom et al., 2012; Kompaníková and Bryja, 2022; Sturrock, 1979). Concurrently, mesenchymal cells derived from the mesoderm begin forming the stroma (Catala, 1998). Around E12-14.5, angiogenesis and signaling from the ChP epithelium lead to complex vascular networks within the stroma (Nielsen and Dymecki, 2010). Maturation of the ChP vasculature from E14.5-16.5 creates a functional blood-CSF barrier (Kompaníková and Bryja, 2022; Liddelow et al., 2012). Embryonic BAMs split into distinct populations by P21 based on their location in the ChP stroma or epithelium (epiplexus) (Van Hove et al., 2019).

The ChP stroma is vascularized by fenestrated capillaries that enable the diffusion of water and small molecules. A single-cell spatial atlas revealed that the stroma contains a plethora of cells, including pericytes, fibroblasts, endothelial cells, immune cells, neurons, and glial cells (Dani et al., 2021). Many immune cells infiltrate from circulation during adulthood, likely through diapedesis across stromal blood vessels (Zhan et al., 2024). The stroma also contains a large number of tissue-resident BAMs. During homeostasis, the static cell bodies of stromal BAMs align with vascular endothelial cells and perform immune surveillance by extending and contracting their processes, allowing them to phagocytose foreign antigens (Shipley et al., 2020). They continue to perform similar functions during inflammation, as evidenced by their elongation around blood vessels after systemic bacterial lipopolysaccharide (LPS) administration (Shipley et al., 2020). Additionally, exposure to circulating blood permits replenishment of the stromal BAM population with infiltrating MHCII^+^CCR2^+^ monocytes, similar to dural BAMs (Van Hove et al., 2019; Goldmann et al., 2016). The high turnover rate of stromal BAMs likely reflects the unique vascular permeability of the ChP stroma, uniquely positioning these cells as immunological gatekeepers that may mediate leukocyte entry and antigen sampling during neurinflammatory conditions (Goldmann et al., 2016; Vara-Pérez and Movahedi, 2025).

Epiplexus BAMs, also known as Kolmer cells, line the apical, ventricle-facing surface of the ChP epithelium. CSF-producing ChP epithelial cells are joined by tight junctions, making this barrier difficult to penetrate (Ghersi-Egea et al., 2018). Therefore, it is hypothesized that epiplexus BAMs primarily retain their embryonic origin and are not replaced by CCR2^+^ blood monocytes under steady state conditions, similar to leptomeningeal and perivascular BAMs (Van Hove et al., 2019; Zhan et al., 2024). Single-cell sequencing shows that steady-state epiplexus BAMs express core microglia gene signatures, like Sall1 and P2ry12 (Van Hove et al., 2019). At E12.5, CD206^+^ cells migrate from the roof plate (early ChP) through the ventricles into the pallium (early cortex) (Hattori et al., 2023). Shared ontogeny and/or regional proximity could explain transcriptomic similarity between microglia and epiplexus BAMs (Van Hove et al., 2019; Hattori et al., 2023). Functionally, high mobility and continuous contact with CSF make epiplexus BAMs excellent at immune surveillance; however, unlike their stromal counterparts, they do not readily phagocytose foreign debris under homeostatic conditions (Shipley et al., 2020). However, under inflammatory conditions—e.g., intracerebroventricular (i.c.v.) injection of LPS—epiplexus BAMs have been shown to migrate to the infection site where they phagocytose debris and immune cell infiltrates, and produce occludin to repair epithelial tight junctions (Xu et al., 2024). Repopulation of epiplexus BAMs with CCR2^+^ monocytes was also observed following this immune insult (Xu et al., 2024). Lehtinen’s group has done a substantial amount of research to understand ChP BAM subtypes, and their findings are summarized in their recent review (Cui et al., 2021).

## 4 The potential role of BAMs in neurodevelopmental disorders

Most of what is known about BAM function comes from adult rodent studies. However, understanding how these cells contribute to neurodevelopment is essential for deciphering their response to early immune insults and their potential involvement in NDDs. In this section, we highlight the current understanding of how prenatal inflammation impacts fetal BAMs and postulate how these cells might contribute to NDD pathology, specifically cortical malformations. We also use neurodegenerative disorders as a lens to predict BAM functions during developmental insults.

### 4.1 Developmental BAMs and corticogenesis

While many studies have documented the functional roles of parenchymal microglia during healthy and disordered neurodevelopment (Cunningham et al., 2013; Matuleviciute et al., 2023; Li and Barres, 2018), much less is known about their border-associated counterparts. Microglia, which directly interact with neurons and other progenitor cells, are known to regulate cortical neuron number and migration, axon outgrowth, synaptogenesis, and synaptic pruning (Thion et al., 2018). In contrast, most BAMs do not directly interface with neurons. Thus, their ability to regulate neurodevelopmental processes, if at all, is likely to be through indirect mechanisms.

In Cui et al. (2020) conducted an MIA study to explore dynamics at the ChP-CSF interface. They found that maternal systemic inflammation, induced by intraperitoneal poly I:C injection at E12.5, led to accumulation of CD68^+^ macrophages in the embryonic ChP epiplexus and elevated CCL2 levels in the CSF at E14.5, demonstrating increased macrophage phagocytic capacity and recruitment of blood monocytes, respectively (Cui et al., 2020). They observed upregulated *Ccl2* gene expression in CSF-facing meningeal tissue; however, they did not investigate these meningeal BAMs. Our group recently identified an increase in the number of fetal leptomeningeal BAMs at E16.5, 7 days after prenatal exposure to maternal influenza A virus infection (Otero et al., 2025). An increase in meningeal BAM numbers was especially prominent above the neocortex, coinciding with our observation of reduced cortical thickness and upper-layer cortical marker, SATB2. Prenatal exposure to mouse cytomegalovirus at E13.5 also resulted in an increase in fetal meningeal macrophages and reduced upper excitatory cortical marker Brn2 in E18.5 embryos (Sakao-Suzuki et al., 2014). Notably, these early life insults occurred around the time brain-resident myeloid cells are trafficking to and taking residence in the developing brain (Utz et al., 2020; Figure 1C). This could highlight a critical developmental window by which prenatal immune insults program brain-resident macrophages, leading to perturbed corticogenesis.

Corticogenesis refers to the creation of the six distinct cortical layers from E11.5 to E16.5 in mice (Mukhtar and Taylor, 2018; Dwyer et al., 2016). Radial glia in the ventricular zone (VZ) contribute to cortical development by acting as guides for migrating neurons and by generating intermediate progenitor (IP) cells in the subventricular zone (SVZ) (Noctor et al., 2004). IPs migrate radially from the SVZ to the marginal zone (MZ), where they establish cortical neurons in embryonic layers I-VI (Dwyer et al., 2016). The mammalian cerebral cortex is responsible for cognition, sensory processing, and motor control, among other functions. Therefore, it is not surprising that cortical abnormalities are a common feature of human NDDs (Khundrakpam et al., 2017; Chiappelli et al., 2017; Goldman et al., 2009; Holiga et al., 2019; Khan et al., 2015; Dong et al., 2018) and MIA animal models (Choi et al., 2016; Otero et al., 2025; Carpentier et al., 2013; Ben-Reuven and Reiner, 2021; Canales et al., 2021). The onset of cortical deficits in NDDs is likely multifactorial and may partly result in changes to surrounding structures, like the meninges and lateral ChP. Indeed, longitudinal studies show increased CSF in the SAS surrounding the cortical surface in infant brains, which preceded their autism spectrum disorder (ASD) diagnosis (Shen et al., 2017, 2013). This coincides with a study that found elevated inflammatory cytokines and chemokines, including CCL2, in the CSF of people with ASD (Vargas et al., 2005). While we are unaware of any clinical study investigating BAM functions in NDDs, it is plausible that these CSF-facing cells (epiplexus and leptomeningeal BAMs in particular) could contribute to heightened inflammatory profiles in the CSF, leading to impaired cortical development. We can further postulate functions of fetal BAMs in NDDs by extrapolating from their known roles in neurodegenerative disorders.

### 4.2 BAM functions during neurodegeneration as a proxy for developmental insults

Several pathological features of neurodegenerative diseases—e.g., pro-inflammatory and pro-phagocytic shifts in resident immune cells, compromised vascular barriers, altered proteostasis—are shared with NDDs. Thus, we propose that known BAM functions during neurodegeneration could be used to predict their responses during developmental insults.

Limited information exists about the role of BAMs compared to microglia during aging and neurodegeneration (Hickman et al., 2018). However, several preclinical studies suggest that BAMs are important cells in the progression and presentation of Alzheimer’s Disease (AD), Parkinson’s Disease (PD), and Multiple Sclerosis (MS) (Sun and Jiang, 2024). For instance, depletion and stimulation of PVMs in a genetic mouse model of mutated amyloid precursor protein (APP) expression (a model of AD) demonstrated that PVMs—not microglia or astrocytes—targeted and cleared amyloid beta aggregates in cortical and leptomeningeal blood vessels (Hawkes and McLaurin, 2009). Another genetic AD mouse model showed that PVMs were responsible for inducing cerebrocortical vascular oxidative stress via reactive oxygen species (ROS) production upon exposure to amyloid beta plaques (Park et al., 2017). However, eliminating PVM expression of CD36, a scavenger receptor protein, or NOX2, an enzyme in the ROS signaling pathway, ameliorated vascular dysfunction (Park et al., 2017; Uekawa et al., 2023). Therefore, PVMs appear to play a complex role in AD pathology, particularly in cortical blood vessel function. Notably, while PVMs develop postnatally, their meningeal origin could give us insight into embryonic leptomeningeal BAM function.

PD and MS preclinical and clinical studies provide insight into both meningeal and perivascular BAM function. Overexpression of alpha synuclein in substantia nigra dopaminergic neurons (a mouse model of PD) increased the number of MHCII^+^ leptomeningeal and perivascular BAMs through the proliferation and infiltration of monocytes (Schonhoff et al., 2023). Conditionally knocking out MHCII in brain-resident macrophages reduced PD-related CD4^+^ T cell infiltration and microglia activation, whereas microglia-specific MHCII deletion had no effect. BAM-specific depletion via i.c.v. clodronate liposome injection recapitulated the CNS-resident macrophage-specific MHCII knockout findings, highlighting BAMs’ role in antigen presentation and immune cell recruitment during inflammatory conditions (Schonhoff et al., 2023). The same study showed that human PD postmortem brains had more CD4^+^ and CD8^+^ T cells near phagocytic CD68^+^ BAMs in the substantia nigra, indicating a similar disease-associated interaction between BAMs and T cells seen in the PD mice (Schonhoff et al., 2023). In EAE (a mouse model of MS), leptomeningeal BAMs and PVMs proliferate during disease onset and present antigens to T cells (Schläger et al., 2016). Leptomeningeal BAMs also produce CCL5 and CXCL9/10/11, which attract and promote adhesion of autoreactive T cells to the meninges (Schläger et al., 2016). Notably, CD163^+^ PVM accumulation has also been observed in the brains of MS patients (Zhang et al., 2011), indicating that BAMs likely play a similar role in recruiting and converting autoimmune T cells in human disease. Interestingly, gene regulation pathways related to antigen presentation were increased in dural BAMs—not leptomeningeal or perivascular BAMs—before the onset of EAE (Min et al., 2024), possibly indicating that this population later contributes to the diseased subdural BAM group. Overall, while several human studies have identified BAM subtypes in diseased tissue (Schonhoff et al., 2023; Zhang et al., 2011), more research is needed to clarify their functions.

Information from neurodegenerative diseases demonstrates the importance of BAMs in immune cell recruitment, vascular function, debris phagocytosis, and antigen presentation during various neuroinflammatory conditions. Notably, their function appears to be largely independent of microglia (Hawkes and McLaurin, 2009; Park et al., 2017; Uekawa et al., 2023; Schonhoff et al., 2023; Schläger et al., 2016). We propose that inflammation during early brain development can alter BAM programming, potentially resulting in aberrant cortical lamination. For instance, meningeal-derived chemokine signaling has been shown to be imperative for proper cortical development (Borrell and Marín, 2006), while exposure to pro-inflammatory cytokines prenatally can disrupt neocortical patterning (Choi et al., 2016). Although these studies did not directly examine BAMs, it is plausible that early life insults can dysregulate BAM signaling, similar to what is reported in neurodegenerative diseases. These altered BAM profiles could contribute to perturbations in cortical lamination either directly through increased production of pro-inflammatory mediators or indirectly by recruiting peripheral immune cells. Indeed, at least one study has demonstrated that culturing human fetal cortical NPCs with inflammatory monocyte-conditioned media impacted cortical neuronal development, enhancing NPC proliferation and reducing neurogenesis (Peng et al., 2008). However, the mechanism by which resident BAMs influence developing cortical neurons remains to be elucidated. Advancements in technology, including the ability to selectively target BAMs, will enable researchers to parse out BAM functions in both homeostatic and inflammatory developmental contexts.

## 5 Tools to manipulate or model BAMs during development

The emerging interest in BAMs necessitates tools that isolate them from microglia. However, they are particularly difficult to study *in utero* due to transient protein expression and overlap with embryonic microglia development. Advances in mouse transgenic models and human 3D culture systems will hopefully help to reveal the exact mechanisms by which embryonic BAMs facilitate neurodevelopment.

Pharmacological inhibitors are common tools to study embryonic brain myeloid cells. CSF1R inhibitors easily cross the BBB and can be administered in rodent chow, thus providing a non-invasive way to examine transient brain myeloid depletion (Paolicelli and Ferretti, 2017; Lei et al., 2020). Liposomal clodronate depletes all brain macrophages by inducing apoptosis once phagocytosed (Van Rooijen and Sanders, 1994). Since liposomal clodronate cannot cross the BBB, it must either be administered *in vitro* or directly into embryonic brains via surgical intervention (Cunningham et al., 2013; Hattori et al., 2020). Interestingly, i.c.v. injection (Schonhoff et al., 2023; Pedragosa et al., 2018; Hawkes and McLaurin, 2009) and intra-cisterna magna (i.c.m.) injection (Drieu et al., 2022) of clodronate liposomes in adult mice depleted BAMs but not microglia. This selectivity has not been evaluated in embryos.

Cre reporter lines, which utilize the Cre-*LoxP* system whereby Cre recombinase targets a gene of interest flanked by *LoxP* sites, are popular genetic tools for studying brain cells. Constitutive *Cx3cr1^Cre^* and inducible *Cx3cr1^CreER^* are among the most widely used transgenic lines for studying brain resident myeloid cells *in utero;* however, *Cx3cr1* recombination occurs in BAMs, microglia, and peripheral monocytes, making it difficult to distinguish cell-specific functions (Goldmann et al., 2016; Brioschi et al., 2023). Furthermore, Cre recombinase fused with estrogen receptor (CreER) prevents enzyme translocation to the nucleus until administration of exogenous ER-binding drug, tamoxifen. While this allows researchers to target specific developmental windows, tamoxifen use during pregnancy leads to adverse offspring outcomes, such as congenital malformations (Lee et al., 2020).

More specific transgenic lines, like *Crybb1*^Cre^, can be used to exclusively study embryonic, non-monocyte-derived macrophages (Brioschi et al., 2023). Like their pharmacological predecessors, these transgenic lines are not ideal for distinguishing BAM from microglia-specific functions. Recent identification of BAM-specific markers has led to the generation of new CreER reporter lines. While CD206 recombination using the *Mrc1*^*creERt*2^ line occurs in meningeal and perivascular BAMs postnatally, *Mrc1* recombination occurs in both BAMs and microglia prenatally because they develop from a common CD206^+^ progenitor (Masuda et al., 2022). Additionally, this line uses tamoxifen for genetic recombination.

Minimally invasive BAM-specific transgenic models are necessary to remove confounding factors caused by concurrent manipulation of microglia/monocytes and by drug-mediated insults. The Split-Cre system, which divides Cre recombinase into two non-functional halves (N- and C-terminal) that reassemble into a functional enzyme only when co-expressed, offers a promising approach to bypass the need for tamoxifen use while maintaining cell-type specificity. A recent study demonstrated that *Lyve1^*ncre*^:Cx3Cr1*^*ccre*^ selectively labels meningeal and perivascular BAMs (Kim et al., 2021). *Pf4^Cre^* is another transgenic line that elicits a high degree of recombination in dural, pial, perivascular, and choroid plexus BAMs (McKinsey et al., 2020). These transgenic lines provide valuable ways to investigate embryonic BAMs without the confounding effects of tamoxifen exposure during pregnancy.

*In vitro* culture and organoid models provide an alternative way to study embryonic BAM function. Human microglia-like cells (iMGs), derived from inducible pluripotent stem cells (iPSCs), have embryonic-like features (Buchrieser et al., 2017). Combining them with regionally patterned organoids (e.g., cortical, choroid plexus, or BBB organoids) could create powerful platforms for studying BAM ontogeny, signaling, and pathology in human developmental contexts (Cerneckis and Shi, 2023). Advances in microfluidic technology, such as brain-on-a-chip (Rodrigues et al., 2024) and ChP-on-a-chip (Lim et al., 2023), may also offer novel insights into how BAMs modulate processes like CSF flow dynamics.

## 6 Conclusion

The cellular and molecular functions of embryonic BAMs in neurodevelopmental homeostasis and disease are still being explored, making this an exciting and rapidly evolving field. While microglia have been the focus of most developmental neuroimmunology research (Antony et al., 2011; Cunningham et al., 2013; Squarzoni et al., 2014; Pont-Lezica et al., 2014; Paolicelli et al., 2011; Zhan et al., 2014; Miyamoto et al., 2016; Loayza et al., 2023; Ozaki et al., 2020; Yu D. et al., 2022; Paolicelli and Ferretti, 2017; Hattori et al., 2020; Coleman et al., 2020), BAMs are located at key brain–immune interfaces and respond dynamically to environmental cues, particularly during inflammatory challenges. Based on a handful of MIA studies and with insights from models of neurodegenerative disorders, we suggest that BAMs might influence cortical development by modulating CSF content, immune cell recruitment, and vascular (re)modeling. Along with our understanding of microglia’s role during development, we can hypothesize additional functions for BAMs (Table 1). However, direct evidence for their specific contributions to neurodevelopment remains limited. Going forward, the development of BAM-specific tools and human-relevant models will be essential to untangle their individual roles. Indeed, while it has been accepted that brain-resident macrophage ontogeny and function are largely evolutionarily conserved, subtle differences between species, such as gestational timing and the *in utero* environment, necessitate human-centric approaches (Bao et al., 2024). A deeper understanding of BAMs’ cellular and molecular functions offers the potential to better understand their influence in NDDs and to discover new therapeutic strategies for early-life neuroimmune regulation.

**TABLE 1:** Brain-resident myeloid cell subtypes during development, health, and disease.

Brain myeloid cell type	Identifying markers	Embryonic brain development	NDDs/MIA	Homeostasis	Neurodegeneration
Microglia	SALL1, TMEM119, P2RY12	NPC proliferation & migration (Swinnen et al., 2013; Antony et al., 2011), axonal fasiculation (Pont-Lezica et al., 2014), dendritic spine formation (Miyamoto et al., 2016)	Interneuron deficits (Yu D. et al., 2022), dysregulated axonal fasiculation (Pont-Lezica et al., 2014), disrupted neurogenesis (Cunningham et al., 2013; Rosin et al., 2021)	Synaptic pruning/neural circuit refinement (Paolicelli et al., 2011; Mosser et al., 2017), immune surveillance (Li and Barres, 2018)	See Hickman et al., 2018
Dural BAMs	MHCII	?	?	Antigen presentation (Min et al., 2024; Rustenhoven et al., 2021), clearance of pathogens/foreign antigens (Rebejac et al., 2022)	Increase in antigen presentation gene regulation pathways in presymptomatic EAE (Min et al., 2024)
Leptomeningeal BAMs	MRC1, LYVE1, CD163	?	Elevated in fetal brains prenatally exposed to IAV (Otero et al., 2025) and CMV (Sakao-Suzuki et al., 2014)	Phagocytosis/scavenging of debris, regulation of CSF flow dynamics (Drieu et al., 2022; Zhan et al., 2024)	Increase in MHCII and antigen presentation in PD (Schonhoff et al., 2023), increased chemokine production/peripheral immune cell recruitment in EAE/MS (Zhang et al., 2011; Schläger et al., 2016)
Perivascular BAMs	MRC1, LYVE1, CD163	Not relevant	?	Phagocytosis/scavenging of debris, antigen presentation (Zhan et al., 2025)	Same as leptomeningeal BAMs plus clearance of amyloid beta aggregates and induction of vascular dysfunction in AD models (Hawkes and McLaurin, 2009; Park et al., 2017; Uekawa et al., 2023)
Epiplexus ChP BAMs	SALL1, P2RY12	?	Accumulation in epiplexus of fetal brains prenatally exposed to poly I:C (Cui et al., 2020)	Immune surveillance (Shipley et al., 2020)	?
Stromal ChP BAMs	MHCII	?	?	Phagocytosis/scavenging of debris/foreign antigens (Shipley et al., 2020)	?

The knowns and unknowns of border-associated macrophage (BAM) subtypes in neurodevelopment, neurodevelopmental disorders (NDDs), homeostasis, and neurodegeneration. Microglia are referenced here to posit potential functional roles of BAMs in development. AD, Alzheimer’s Disease; CMV, cytomegalovirus; CSF, cerebrospinal fluid; EAE, experimental autoimmune encephalomyelitis; IAV, influenza A virus; MIA, maternal immune activation; MS, Multiple Sclerosis; NPC, neural precursor cell; PD, Parkinson’s Disease.
